# Prevalence of viral sexually transmitted infections and HPV high-risk genotypes in women in rural communities in the Department of La Paz, Bolivia

**DOI:** 10.1186/s12879-020-4931-1

**Published:** 2020-03-06

**Authors:** Marianela Patzi-Churqui, Katty Terrazas-Aranda, Jan-Åke Liljeqvist, Magnus Lindh, Kristina Eriksson

**Affiliations:** 1 Department of Rheumatology & Inflammation Research, Institute of Medicine, Sahlgrenska Academy, Gothenburg University, Box 480, S-405 30 Gothenburg, Sweden; 2 Unidad de Virología, Inmunidad e Infección, Insituto SELADIS, Facultad de Ciencias Farmacéuticas y Bioquímicas, Universidad Mayor de San Andrés, La Paz, Bolivia; 3 Department of Infectious Diseases/Virology, Institute of Biomedicine, Sahlgrenska Academy, Gothenburg University, Gothenburg, Sweden

**Keywords:** Prevalence, Sexually transmitted infections, Women, La Paz, Bolivia, Rural communities, HSV-2, HIV, High-risk HPV, HBV

## Abstract

**Background:**

Bolivia has the highest prevalence of cervical cancer in South America and the prevalence of viral sexually transmitted infections (STIs) among people in urban cities is increasing. Little is known about the prevalence of viral STIs in rural communities, which generally have limited access to health care. In order to study the prevalence of viral STIs in rural Bolivia, we recruited women from villages and towns in the Department of La Paz in Bolivia.

**Methods:**

Three hundred ninety-four female participants were assessed for IgG-antibodies to herpes simplex virus type 2 (HSV-2), human immunodeficiency virus (HIV) and hepatitis B virus (HBV, anti-HBc), as well as for the presence of HBV surface antigen (HBsAg) in dried blood spots. The prevalence of 12 high-risk types of human papillomavirus (HPV) was assessed by qPCR in dried cervicovaginal cell spots from 376 of these women. χ^2^ test was used to compare variables between the populations and binary logistic regression was used to identify risk factors associated with the positivity of the tests.

**Results:**

The seroprevalence of HSV-2 was 53% and of HBV 10.3%. HBAg was detected in 15.8% of women with anti-HBV antibodies indicating chronic infection. The frequency of high-risk HPV infection was 27%, with the most prevalent high-risk HPV types being HPV 56, 39 and 31 followed by HPV 16 and 18. Finally, none of the 394 women were seropositive for HIV, and about 64% of the studied population was positive for at least one of the viral infections.

**Conclusions:**

Women in Bolivian rural communities in La Paz show a high prevalence of HBV, HPV and, in particular, HSV-2. In contrast, none of the women were HIV positive, suggesting that the HIV prevalence in this population is low. The pattern of high-risk HPV types differed from many other countries with a predominance of HPV-types not included in the Gardasil vaccine which was officially introduced in Bolivia in April 2017.

## Background

To investigate and report the prevalence of viral sexually transmitted infections (STIs) other than HIV is important for the introduction or improvement of a wide range of health policies and programs in all regions. It is known that more than 30 microorganisms are sexually transmitted including herpes simplex virus type 2 (HSV-2), hepatitis B virus (HBV), human papillomavirus (HPV) and human immunodeficiency virus (HIV). Estimations according to the World Health Organization (WHO) indicate that more than 1 million STIs are acquired every day.

Almost 300 million people are infected with HPV, which if becoming persistent may cause cervical cancer [1]. More than 400 million individuals are estimated to be infected with HSV-2 which causes genital herpes [2]. Approximately 250 million people have chronic HBV infection which can cause liver cirrhosis and hepatocellular carcinoma [3]. According to the Joint United Nations program on HIV/AIDS (UNAIDS), approximately 37 million people are living with HIV which may cause acquired immune deficiency syndrome (AIDS). These general numbers are based on reports and studies performed around the world. However, the prevalence of viral STIs is unknown in rural areas in Bolivia where the majority of indigenous people are settled.

Bolivia has a population of around ten million people and 40 to 70% are self-identified as indigenous peoples [4]. Although new policies have been introduced to improve the general health programs, Bolivia still has the highest rates of cervical cancer in South America with a yearly incidence of 38.5/100,000 and a mortality rate of 18.2/100,000 according to the Information Centre of HPV and Cancer Catalan Institute of Oncology (ICO). Globally, the major risk factor for developing cervical cancer are HPV types 16 and 18 [5]. An ongoing cytology-based screening program for all women was introduced in Bolivia in 2006, but it reaches mostly urban areas and has a poor coverage that did not exceed 17% for Pap smear test and 20% for visual inspection under acetic acid [6]. Unfortunately 50 to 80% of the screened women do not attend the follow-up appointments [7], and there is no system of quality control and assurance of diagnoses. Therefore, neoplasia caused by persistent HPV-infection is rarely detected during early stages of disease but most often occur at diagnosis of cervical cancer and thereby barely curable. A vaccine targeting HPV 16, 18, 6 and 11 was however introduced in Bolivia in April 2017 and this program will reduce the mortality rates of cervical cancer. The vaccine is given to school-girls aged 10–12 years and the coverage of the vaccination was 88% in 2017 and 61% in 2018 [8].

HSV-2 infection is a risk factor for the acquisition of HIV in people who practice unsafe sex, and it is known that the virus is more prevalent in women compared to men. In 2012 the general seroprevalence of HSV-2 of people aged 15–49 years in the Americas is estimated to be 14.4%, while the highest world-wide prevalence is in African populations with an overall estimated prevalence of 31.5% [2].

The Amazonas is an area of high endemicity of HBV, with reported prevalences ranging from 8 to 25% [9]. Countries in South America, including Bolivia, have introduced general childhood vaccination against HBV, leading to a decreased incidence. However, in Bolivia the vaccination coverage rate is still one of the lowest in South America [10], and its impact on HBV prevalence in rural areas is uncertain.

In 2017, UNAIDS reported that about 21,000 people are living with HIV in Bolivia with a general prevalence of 0.3%, and that only 36% of those infected are on treatment. A later report indicates an increase of HIV incidence [11]. The general knowledge regarding disease transmission and treatment is low in urban areas [12], and probably even lower in rural communities.

In rural areas the poor population have limited information about STIs, little/no access to the national health care system, and no resources for treatment. This study therefore seeks to obtain data about the prevalence and possible risk factors of viral STIs in women living in rural areas on the way to the Amazonas of the Department of La Paz in Bolivia, which will help to improve the screening programs and health policies.

## Methods

### Study population and collection of specimens

Our study population was recruited in the rural provinces of Abel Iturralde (the lowlands of the Amazonas at altitudes of 150 to 600 m above sea level) and Caranavi (the adjacent lower highlands at 1000 m above sea level) from the Department of La Paz in Bolivia during five field trips; three in the Abel Iturralde province during the dry seasons (July to October) between 2015 and 2017 and two in Caranavi in March 2018 and May 2019. Our research group, a unit working at the Universidad Mayor de San Andrés (UMSA), visited the three villages Tumupasa, San Silvestre and Santa Rosa de Maravilla (mostly indigenous population below 1200 inhabitants) [13], the small town San Buenaventura (population approximately 8000) and the large town Caranavi (population of 50,000) and spread information through the local authorities explaining our objectives and the benefits of our study.

Participants were recruited either by direct invitations to small mother groups, unions, local authorities and indigenous leaders or via general invitations by radio and television. The examination and collection of samples was done in the nearest local health center or hospital by laboratory technicians from UMSA. Women who were menstruating or were more than 12 weeks pregnant were excluded from the study. Eligible participants (*N* = 394) received extended oral and written information regarding STIs and signed an informed consent form. In the case of women under the age of 18 informed consent was signed by their guardians. Demographic data was collected through a questionnaire and a personal interview. Blood samples (*N* = 389) and cervicovaginal cell samples (*N* = 376) were collected from female volunteers.

Because of the lack of sanitary services (including electricity) in some of these areas, sampling was performed using filter papers that preserve the biological material and allowed the transportation and assessment of the samples. Whole blood samples were therefore collected as Dried Blood Spots (DBS) in Whatman 903 filter papers (Protein Saver™ 903 Card) for the evaluation of antibodies against HSV-2, HBV and HIV as well as for HBV antigen. Exfoliated cervicovaginal cells were collected by swabbing the lateral walls using sterile cotton swabs that were then transferred in 1 mL of essential D-MEM medium (Sigma–Aldrich, MO, USA) and kept at 4 °C. At the laboratory in La Paz city the samples were centrifuged at 1500 rpm for 10 min at 4 °C and suspended cells in 150 μL of medium were transferred and applied on 2 × 2.5 cm Nucleic-card color matrix spots (NUCLEIC-CARD Thermo Fisher Scientific, USA) as dried cervicovaginal cell spots (DCCS). DBS and DCCS were kept at − 20 °C until their further transport to and analysis in Sweden. Data was managed anonymously according to the ethical permission CEI-UMSA 0215 obtained from the Ethical committee at UMSA, La Paz, Bolivia.

### Laboratory methods

#### Elution of DBS

Blood proteins were extracted from DBS by punching one bloodstained circle of 5 mm diameter from the filter paper as previously described [14, 15], The spots were soaked for 24 h at 4 °C in 300 μL of diluent buffer. The papers were removed and the elutions were centrifuged at 4500 rpm for 10 min. A total volume of 250 μL of elution was obtained from each spot and either assessed immediately (see below) or kept at − 20 °C until analysis.

#### Extraction of DNA from the DCCS

DNA was extracted and purified from DCCS using QIAGEN mini kit as described [16], One spot of 2 × 2.5 cm was placed in 600 μL of sterile PBS buffer in a 1.5 mL tube for 1 h at 4 °C. After that, 400 μL of the supernatant was collected in a different tube in order to do the lysis and the extraction of genomic DNA according to the manufacturer’s instructions. About 150 μL of eluted purified DNA was obtained and quantified by using a Nanodrop N-1000 instrument. DNA samples were stored at − 20 °C until assessment.

#### Serology

Elutions of DBS were used to assess antibodies for HSV-2, HBV, and HIV using commercial ELISA kits; HSV-2 (Herpeselect2, FOCUS Diagnostics), anti-HBV anti-core antibodies (Murex anti-HBc total, Diasonin S.p.A. UK Branch), and HIV (Murex HIV-1.2.0, Diasonin S.p.A. UK Branch). In addition, we measured the presence of HBV surface antigens in eluted DBS using ELISA (Murex HBsAg Version3, Diasonin S.p.A. UK Branch). As the dried blood samples contain lysed red blood cells, which can interfere with ELISA readout and give false-positive results, we increased the cut-off values provided by the manufacturers of the ELISA kits by multiplying with a factor of 1.5 [17, 18].

Equivocal and low positive samples for HSV-2 by FOCUS ELISA were confirmed with the detection of antibodies to the mature portion of glycoprotein G-2 using Western blot as described [19]. Briefly, HSV-2 antigens were subjected to polyacrylamide gel electrophoresis under reducing conditions by using NuPAGE 7% Tris-acetate gels (Novex). Antigens were electrotransferred to a membrane (Milipore Corp.) and the strips were incubated overnight with eluted samples at a 1:3 dilution. Peroxidase-labeled rabbit anti-human IgG (DAKO) was used as conjugate and 4-chloro-1-naphthol was used as substrate to visualize binding. A positive profile was defined as reactivity to mgG-2 (~ 120 kDa).

#### HPV typing

Purified DNA from DCCS were assessed by a Taqman real-time PCR assay which identifies 12 high-risk (16, 18, 31, 33, 35, 39, 45, 51, 52, 56, 58 and 59) and two low-risk (6 and 11) types, by targeting segments of the E6/E7 region [20]. The amplification was performed in 8 duplex real-time PCR reactions, including the 14 HPV types and the human gene (the β-globin gene) which served as a control for sample quality and overall PCR efficiency. The PCR was run for 45 cycles (15 s at 95 °C, 60 s at 58 °C) on an ABI 7500 instrument (Applied Biosystems). Serial dilutions of pUC57 plasmids containing target segments of each HPV type, sized 82–134 base pairs (synthesized by GenScript Corp.) were used to verify a high PCR efficiency. Only samples yielding at a cycle threshold (C_T_) for β-globin below of 37 were included in the analysis.

#### Statistical analysis

Descriptive data are presented as percentages, median, prevalence estimates and 95% confidence intervals (CIs). Statistical analyses were calculated using χ^2^ test comparing among groups using PRISM 7.0® from GraphPad Software Inc., San Diego, CA. A *p* value below 0.05 was considered statistically significant.

The associations between the dependent variable (viral STI) and the independent variables; age, occupation, number of children, current family planning methods, residence and coinfections were tested by binary logistic regression. This analysis was used to estimate crude and adjusted odds ratios and 95% CIs for each category compared to a reference category (Ref). Variables or categories that showed association with the outcome at *p* < 0.1 were included in the adjusted models as in other studies [21]. Data analysis were performed using SPSS 24.0 IBM, Chicago, USA.

## Results

### Demographic data of the study population

We investigated the prevalence of viral STIs in women from two provinces of the Department of La Paz, which are inhabited mainly by rural and semi-rural indigenous population. Demographic data was taken from the 394 participants through a questionnaire and an interview. The study population (Table 1) was divided in three groups: villages, a small town and a large town. The median age was 34. The median number of children was 2 (0–10), and women living in villages tended to have more children than women living in towns. The majority of the participants were housewives (51.5%), which were particularly common in villages. 52.5% of the women did not use any family planning method, and just 14 women (3.5%) reported the use of condoms. The majority of women 271 (69%) reported to have had a cytological inspection; however 62% (167/271) did not know the results of the examination (data not shown).

**Table 1:** Demographic characteristics of the participants according to residence

				Groups						
	Total			Villages		Small town		Large town		
	(***n*** = 394)			(***n*** = 77)		(***n*** = 73)		(***n*** = 244)		
	N	(%)	95% CI	N	(%)	N	(%)	N	(%)	***P*** value^**1**^
Age (median = 34)										0.95
< 26	98	(25)	21–29	17	(22)	19	(26)	62	(25)	
26–34	105	(27)	22–31	24	(31)	20	(27)	61	(25)	
35–45	100	(25)	21–30	19	(25)	18	(25)	63	(26)	
> 45	91	(23)	19–28	17	(22)	16	(22)	58	(24)	
Occupation										< 0.0001
Housewife	203	(51.5)	47–56	63	(82)	41	(56)	99	(40.5)	
Self employed^2^	138	(35)	30–40	5	(6)	26	(36)	107	(44)	
Professional or student	53	(13.5)	10–17	9	(12)	6	(8)	11	(15.5)	
Number of children (median = 2)										< 0.0006
0	41	(10)	8–14	9	(12)	6	(8)	26	(11)	
1–3	229	(58)	53–63	30	(39)	38	(52)	161	(66)	
4–10	124	(32)	27–32	38	(49)	29	(40)	57	(23)	
Current family planning method										0.59
None	207	(52.5)	48–57	45	(58)	40	(55)	122	(50)	
Hormonal contraception	130	(33)	28–38	27	(35)	22	(30)	81	(33)	
Others^3^	43	(11)	8–14	3	(4)	10	(14)	30	(12)	
Condom	14	(3.5)	2–6	2	(3)	1	(1)	11	(5)	
Cytological inspection PAP										0.2
Ever	273	(69)	64–73	46	(60)	50	(68)	177	(72)	
Never	121	(31)	27–36	31	(40)	23	(32)	67	(28)	

^1^*P* value was calculated using χ^2^ test between residence groups

^2^Self-employed: Merchant, artisan, cook and farmer

^3^Others: Intra uterine devise (IUD), tubal ligation and calendar

### Seroprevalence of HSV-2

The seroprevalence of HSV-2 was assessed by two methods; all samples were first analyzed using an HSV-2-specific ELISA. Equivocal and low positive samples were confirmed using the “gold standard”, western blot. More than 74% of the samples were positive for HSV-2-specific antibodies using ELISA, but after confirmation using western blot the prevalence was determined to 53% (Table 2). The seroprevalence of HSV-2 increased with age and in women with many children, also when adjusted for age (Table 2). Overall, HSV-2 infection was more common in women living in villages as compared to women living in small and large towns, and a similar association was found after adjustment for age.

**Table 2:** Seroprevalence of HSV-2 by demographic characteristics of the total population

HSV-2									
Variable group	**Total**	**Positive**		**Crude**			**Adjusted**		
	**N**	**N**	**(%)**	**OR**	**95% CI**	**P value**^1^	**OR**^**2**^	**95% CI**	***P*value**
All women	389	205	(53)		47.7–57.6				
Age									
< 26	98	36	(37)	1.0	(Ref)				
26–34	104	57	(55)	2.1	**1.2–3.6**	**0.01**			
35–45	98	64	(65)	3.2	**1.8–5.8**	**0.0001**			
> 45	89	48	(54)	2.0	**1.1–3.6**	**0.019**			
Occupation									
Professional or student	51	21	(41)	1.0	(Ref)		1.0	(Ref)	
Self employed^3^	136	65	(48)	1.3	0.7–2.5	0.40	0.9	0.5–1.9	0.90
Housewife	202	119	(59)	2.0	**1.1–3.9**	**0.02**	1.6	0.8–3.1	0.10
Number of children									
0	41	11	(27)	1.0	(Ref)		1.0	(Ref)	
1–3	225	114	(51)	2.8	**1.3–5.9**	**0.006**	2.7	**1.2–5.8**	**0.01**
4–10	123	80	(65)	5.0	**2.3–11**	**0.0001**	4.6	**1.8–11**	**0.001**
Current family planning method									
Condom	14	4	(29)	1.0	(Ref)		1.0	(Ref)	
Hormonal contraception	128	74	(58)	3.4	**1.0–11**	**0.04**	2.6	0.8–8.9	0.40
Others^4^	41	25	(61)	3.9	**1.0–14**	**0.04**	2.5	0.6–8.9	0.10
None	206	102	(49)	2.4	0.7–8.1	0.14	1.7	0.5–5.9	0.20
Residence									
Large size town	239	112	(47)	1.0	(Ref)		1.0	(Ref)	
Small size town	73	41	(56)	1.4	0.8–2.4	0.16	1.5	0.8–2.5	0.15
Villages	77	52	(67)	2.4	**1.4–3.9**	**0.0016**	2.4	**1.4–4.1**	**0.02**

^1^*P* value was calculated between the subgroups or variables compared to the reference variable

^2^Odds ratio adjusted for age

^3^Self-employed: Merchant, artisan, cook and farmer

^4^Others: Intra uterine devise (IUD), tubal ligation and calendar

Abbreviations: *OR*: odds ratio, *CI* Confidence interval, *Ref* Reference

### Seroprevalence of HBV

Thirty-eight women (out of 389 tested) had HBV core-specific antibodies. Six of these 38 women (i.e. 15.8%) also had detectable HBsAg in their blood indicating a chronic infection. In addition, we found two acute cases of HBV infection, i.e. women that had detectable HBsAg in their blood but no antibodies. Having in total 40 cases, the prevalence for HBV was 10.3% (Table 3). There were no statistically significant differences in age, occupation, number of children, contraceptive use or site of residence between HBV-positive and HBV-negative women (Table 3). It should be noted that the women included in this study were born before the introduction of the HBV vaccination program of children.

**Table 3:** Seroprevalence of anti-HBVc and HBAg by demographic characteristics of the total population

Anti-HBVc									
Variable group	**Total**	**Positive**		**Crude**			**Adjusted**		
	**N**	**N**	**(%)**	**OR**	**95% CI**	***P*value**^**1**^	**OR**^**2**^	**95% CI**	***P*value**
All women	**389**	38	(9.7)		7.1–13				
HBAg	**8**	6	(15.8)		7.1–30				
Age									
< 26	98	6	(6)	1.0	(Ref)				
26–34	104	10	(9.6)	1.6	0.6–4.6	0.36			
35–45	98	12	(12)	2.1	0.8–5.9	0.14			
> 45	89	10	(11)	1.9	0.7–5.6	0.21			
Occupation									
Professional or student	51	3	(4)	1.0	(Ref)		1.0	(Ref)	
Self employed^3^	136	17	(12)	3.5	0.8–16	0.10	3.0	0.6–14	0.17
Housewife	202	19	(9.4)	2.5	0.5–11	0.22	2.2	0.5–10	0.30
Number of children									
0	41	1	(2)	1.0	(Ref)		1.0	(Ref)	
1–3	225	22	(10)	4.3	0.6–33	0.16	4.0	0.5–31	0.19
4–10	123	15	(12)	5.5	0.7–43	0.10	4.5	0.5–40	0.17
Current family planning method									
Condom	14	1	(7)	1.0	(Ref)		1.0	(Ref)	
Hormonal contraception	128	13	(10)	1.5	0.2–12	0.72	1.1	0.1–9.6	0.72
Others^4^	41	3	(7)	1.0	0.1–11	0.98	0.7	0.1–7.5	0.74
None	206	21	(10)	1.5	0.2–11	0.71	1.0	0.1–11	0.98
Residence									
Large size town	239	29	(12)	1.0	(Ref)		1.0	(Ref)	
Small size town	73	5	(7)	0.5	0.2–1.4	0.20	0.5	0.2–1.4	0.20
Villages	77	4	(5)	0.4	0.1–1.1	0.09	0.4	0.1–1.1	0.09

^1^*P* value was calculated between the subgroups or variables compared to the reference variable

^2^Odds ratio adjusted for age

^3^Self-employed: Merchant, artisan, cook and farmer

^4^Others: Intra uterine devise (IUD), tubal ligation and calendar

Abbreviations: *OR* Odds ratio, *CI* Confidence interval, *Ref* Reference

### Prevalence of high-risk HPV

**Fig. 1:**
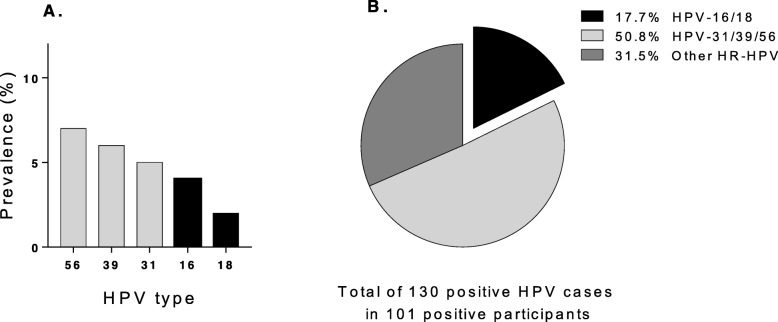
Prevalence of Human Papilloma Virus (HPV) genotypes in cervical samples from 101 participants with detected HPV infection. Measurement of DNA by qPCR of cervical swabs samples eluted. **a**. Prevalence of the five most prevalent HPV positive types found around 376 participants. **b**. Distribution of HPV 16/18, HPV 31/39/56, and other high-risk (HR-HPV) found in 101 samples

Purified DNA from 379 DCCS was analyzed by PCR for the detection of 12 high-risk (HR-HPV) and 2 low-risk (LR-HPV). Three of the DCCS were excluded as no DNA (viral or human) was detected. For the remaining 376 samples, 27% were positive for at least one high-risk HPV type (Table 4). Women below 26 years-of-age showed the highest prevalence (37%) (Table 4). There were no statistically significant differences in age, occupation, number of children, contraceptive use or site of residence between HPV-positive and HPV-negative women (Table 4), nor was there any association between HPV infection and seroprevalence to HSV-2 or HBV. Quite surprisingly, infection with HPV 16 and 18 was relatively rare with a prevalence of only 4 and 2% respectively (Fig. 1a), and only accounted for 17.7% of the total high-risk HPV infections (Fig. 1b). Instead, the most common high-risk types found were HPV 31, 39 and 56, which together accounted for more than 50% of the identified high-risk HPV (Fig. 1b). Multiple HR-HPV infections were detected in 24.8% (25/101) of the HPV-infected women (supplementary Table 1), which means that 6.6% of all investigated women had multiple ongoing high-risk HPV infections. The LR-HPV type 6 and 11 were detected in 8 women, 6 of whom were also positive for at least one high-risk HPV type (supplementary Table 1).

**Table 4:** Prevalence of High risk HPV by demographic characteristics of the total population

HR-HPV									
Variable group	Total	Positive		Crude			Adjusted		
	**N**	**N**	**(%)**	**OR**	**95% CI**	***P*value**^**1**^	**OR**^**2**^	**95% CI**	***P*value**
All women	376	101	(27)						
Age									
< 26	96	35	(37)	1.0	(Ref)				
26–34	98	21	(21)	0.5	**0.2–0.9**	**0.02**			
35–45	96	23	(24)	0.5	0.3–1.0	0.06			
> 45	86	22	(26)	0.6	0.3–1.1	0.10			
Occupation									
Professional or students	53	18	(34)	1.0	(Ref)				
Self employed^3^	134	35	(26)	0.7	0.3–1.4	0.28	0.8	0.4–1.7	0.56
Housewife	189	48	(25)	0.7	0.3–1.3	0.22	0.7	0.4–1.5	0.40
Number of children									
0	41	14	(34)	1.0	(Ref)				
1–3	218	64	(29)	0.8	0.4–1.6	0.50	0.8	0.4–1.6	0.60
4–10	117	23	(20)	0.5	0.2–1.0	0.06	0.5	0.2–1.0	0.15
Current family planning method									
Condom	14	3	(21)	1.0	(Ref)				
Hormonal contraception	126	29	(23)	1.1	0.3–4.2	0.89	1.4	0.3–5.4	0.64
Others^4^	41	12	(29)	1.5	0.4–6.4	0.57	2.2	0.5–10	0.29
None	195	57	(29)	1.5	0.4–5.6	0.53	2.0	0.5–7.6	0.31
Cytological inspection PAP									
Ever	265	68	(26)	1.0	(Ref)				
Never	111	33	(30)	1.2	0.7–2.0	0.40	1.2	0.7–2.0	0.50
Residence									
Large size town	239	70	(29)	1.0	(Ref)				
Small size town	68	14	(21)	0.6	0.3–1.2	0.20	0.6	0.3–1.2	0.20
Villages	69	17	(25)	0.8	0.4–1.4	0.50	0.8	0.4–1.4	0.40
Coinfection									
HSV-2 Negative	179	45	(25)	1.0	(Ref)				
HSV-2 Positive	192	55	(29)	1.2	0.7–1.9	0.40	1.2	0.8–2	0.30
HBAg Negative	363	94	(50)	1.0	(Ref)				
HBAg Positive	8	4	(26)	2.7	0.7–11	0.10	2.7	0.7–11	0.20

^1^*P* value was calculated between the subgroups or variables compared to the reference variable

^2^Odds ratio adjusted for age

^3^Self-employed: Merchant, artisan, cook and farmer

^4^Others: Intra uterine devise (IUD), tubal ligation and calendar

Abbreviations: *OR* Odds ratio, *CI* Confidence interval, *Ref* reference

### Prevalence of co-infections

The number of viral STI in the 371 women where all ongoing viral evaluations were performed (i.e. also HPV) was assessed and is summarized in (Table 5). None of the women had antibodies to HIV (data not shown). 36% of the participants were negative for all four viral infections. 48% were positive for one viral STI, 16% for two viral STIs, and 0.5% for three viral STIs. The majority of co-infections were due to HSV-2 and HPV.

**Table 5:** Presence of single, multiple or no viral STIs in 371 participants

Number of infections	Pathogens	No.	(%)	Total	(%)	95% CI
0	No infections	133	(35.8)	133	**(35.8)**	31.1–40.8
1	HPV	43	(11.6)	178	**(48)**	42.9–53.0
	HSV-2	134	(36.1)			
	HBsAg	1	(0.3)			
2	HSV-2 + HBsAg	3	(0.8)	58	**(15.6)**	12.3–19.7
	HPV+ HBsAg	2	(0.5)			
	HSV-2 + HPV	53	(14.3)			
3	HSV-2 + HPV + HBsAg	2	(0.5)	2	**(0.5)**	0.1–1.9

## Discussion

In Bolivia, the prevalence of viral STIs has increased during the last 10 years, but this has mainly been investigated in larger towns and cities. In the present study, we assessed the presence of viral STIs in 394 women living around villages and towns in the north of the Department of La Paz and found that the burden of HSV-2, HBV and HPV is higher than in larger cities.

The HSV-2 seroprevalence varies between South America and Caribbean countries and relate to behavioral and social conditions. It increases with age [2], and was in the present study of a rural female population (53%) compared to the overall prevalence in women in the Americas which is estimated to be 14.4%. Our study confirmed the previous observations that the prevalence of HSV-2 increased with age and number of children. It was higher than what has been found in rural places of for example Haiti and Costa Rica where reported prevalences are 42 and 38%, respectively [22, 23], and similar to prevalences in Durban, Tanzania, and a region in Brazil [19, 24, 25]. Our observation that HSV-2 was more prevalent in small villages compared to the small and large towns differs from what has been observed in Haiti, Tanzania and Brazil where the prevalence of HSV-2 was higher in urban compare to rural areas. However, studies in Australia showed that the HSV-2 prevalence is higher in indigenous people [26].

In female African populations, acquisition of HSV-2 infection is associated with risk factors such as prostitution, limited access to health care, and unnegotiable unsafe sex with their partners due to economic dependence [27]. We have no information regarding possible prostitution in our study population, but these women have a very limited access to health care or health information. In addition, Bolivian men from certain indigenous populations show a high rate of infidelity [28] and the vast majority of the women in this study reported unprotected sex with their partner. Because of cultural reasons, we were not able to ask about the number of sexual partners to all participants; however, women with only one sexual partner have high risk of acquiring STIs, due to the scarcity of condom use, which is 4% in this study [29]. Prolonged use of hormonal contraception can be considered a risk factor to acquire a genital infection [30]. We did not assess the longevity of hormonal contraceptive use so we could not confirm this in our study population.

Twenty-seven percent of the women in this study had an ongoing infection with one or several HR-HPV. This is higher than the 8 to 18% previously reported in rural and urban regions of Bolivia, which indicates that the prevalence of HPV in rural north of La Paz might be higher than in other regions of Bolivia [31–33]. HPV infection was most common in younger women, which is in accordance with previous studies [34]. This study did not find a significant difference or association between the number of children and the infection of HPV, but 19% of women positive for HPV that have more than four children are at high risk to develop squamous-cell cancer [35], if they are not screened and treated.

The 12 high risk HPV types we analyzed are the most important ones due to their association with the development of cervical cancer. These genotypes represent the class I carcinogens for cervical cancer according to the (WHO/IARC), and the most common high-risk HPV types we identified were HPV 56, 39 and 31. Previous studies from Bolivian small towns have identified HPV 31 and 58 as the most common HPV high-risk types [31] while in cities HPV 16, 31, 51 and 58 [32], were most common. This indicates that the pattern of HPV high-risk types for cervical cancer may vary between different regions [36], and also Western Europe [37], where HPV 16 and 18 predominate. This is an important finding because the studies conducted in Bolivian cities have supported the introduction of the HPV 16/18/6/11 vaccine in Bolivia. Fortunately, the vaccine confer some cross protection to the HPV types 31, 33 and 45, which are genetically related to 16 and 18 [38], but it is not known if the vaccine protects against HPV 39 and 56.

Almost 10% of the women in this study had antibodies to HBV, which is similar to the 8% reported in other South American countries such as Colombia and Brazil, and thus confirms the high seroprevalence of HBV in the Amazonas region [9, 39]. Native Bolivians living in neighboring Department in close proximity to the Amazonas have an even higher HBV burden with a seroprevalence of 27.5% [40]. In our study, 16% of those with HBV-specific antibodies were also positive for HBsAg indicating a chronic infection. Thus, we estimate that 2% of women living in these rural areas are chronic HBV carriers. The HBV vaccination that was introduced in Bolivia in year 2000 has fortunately reduced the HBV prevalence in younger age groups [10].

No positive cases for HIV was found, which is in agreement with the low estimates by UNAIDS with only a 0.3% of prevalence in adults aged 15 to 49 years, and zero cases from a study of 885 healthy women mostly urban populations in Bolivia [12]. The program for HIV/AIDS in Bolivia show a higher prevalence in major cities such as Santa Cruz, Cochambamba and La Paz with a total prevalence of 1.3, 1, and 0.3%, respectively in pregnant women [41], with an increase of new cases [11]. Overall, the prevalence of HIV is low in Bolivia compared to e.g. Brazil, which is interesting given the high prevalence of HSV-2 as well as of unprotected sex, both of which are major risk factors for HIV transmission.

Almost 70% of the rural women included in this study were positive for at least one of the four viral STIs. Approximately 15% of the women had more than one viral STI, and the majority of these co-infections involved HSV-2 and HPV. We did not find a positive association between HSV-2 and HPV infections indicating that these infections do not predispose for each other. However, several studies show that HSV-2 infection is an important risk factor for the development of invasive cervical cancer in HPV-infected women [42–44]. Thus, the high incidence of HSV-2, particularly in women living in rural villages, might represent one underlying mechanism for the high incidence of cervical cancer in Bolivian women.

There are certain limitations to this study. First, STIs are stigmatizing in which might have generated a bias in the recruitment process. Second, many women from villages were afraid to participate because they reported that the Pap smear test was painful, and many of them have never encounter a cytological inspection by medical staff. Third, due to the high rate of illiteracy we could only collect data through oral interviews which might have produced some bias in our data.

## Conclusion

We conclude that there is an unusually high prevalence of HSV-2 infection in women from villages and towns in the Department of La Paz in Bolivia, and that both HPV and HBV infections are common. In addition, we note that the pattern of high-risk HPV types differ both from what is found in Europe and included in the HPV vaccine introduced in Bolivia in 2017.

## Supplementary information

**Additional file 1 Table 1.** Types of human papilloma virus detected in cervical samples of 103 participants with HR-HPV and LR-HPV.

## Data Availability

The data used and/or analyzed during the current study available from the corresponding author on reasonable request.

## Abbreviations

AIDS: Acquired immune deficiency syndrome
Anti-HBc: Hepatitis B viral core antibodies
CI: Confidence interval
CIMTA: Consejo Indigena de Mujeres Tacana
CIPTA: Consejo Indigena del pueblo Tacana
DBS: Dried blood spots
DCCS: Dried cervicovaginal cell spots
DNA: Deoxyribonucleic acid
ELISA: Enzyme-linked immunosorbent assay
HBsAg: Hepatitis B viral surface antigen
HIV: Human immunodeficiency virus
HPV: Human papilloma virus
HR-HPV: High risk-human papilloma virus
HSV-2: Herpes simplex virus type 2
IARC: International agency for research on cancer
ICO: Information centre of HPV and cancer catalan institute of oncology
LR-HPV: Low risk-human papilloma virus
OR: Odds ratio
PBS: Phosphate-buffered saline
PCR: Polymerase chain reaction
SELADIS: Instituto de Servicios de Laboratorio de Diagnóstico e Investicagión en Salud
SIDA: Swedish international development cooperation agency
STIs: Sexually transmitted infections
UMSA: Universidad Mayor de San Andrés
UNAIDS: Joint united nations program on HIV/AIDS
WHO: World health organization
