# Supplementary material for: Prevalence of viral sexually transmitted infections and HPV high-risk genotypes in women in rural communities in the Department of La Paz, Bolivia
Source: BMC Infect Dis. 2020 Mar 6;20:204. doi: 10.1186/s12879-020-4931-1 (PMC7060520; doi:10.1186/s12879-020-4931-1)
Supplement: Supplementary file 1 — Additional file 1 Table 1. Types of human papilloma virus detected in cervical samples of 103 participants with HR-HPV and LR-HPV. [file 12879_2020_4931_MOESM1_ESM.docx]

**Supplementary table 1.** Types of human papilloma virus detected in cervical samples of 103 participants with HR-HPV and LR-HPV.

|  |  | **HPV types detected** | |
| --- | --- | --- | --- |
|  | **Case**  **number** | **High risk** | **Low risk** |
|  | 1 | 31 |  |
|  | 2 | 39 |  |
|  | 3 | 56 |  |
|  | 4 | 51 |  |
|  | 5 | 39, 56 | 6 |
|  | 6 | 39, 45 |  |
|  | 7 | 39, 45 |  |
|  | 8 | 56 |  |
|  | 9 | 58 |  |
|  | 10 | 31 |  |
|  | 11 | 18, 31, 59 |  |
|  | 12 | 52 |  |
|  | 13 | 31, 39 |  |
|  | 14 | 16 |  |
|  | 15 | 39, 52 |  |
|  | 16 | 16 |  |
|  | 17 | 39, 51 |  |
|  | 18 | 39 |  |
|  | 19 | 16, 18 |  |
|  | 20 | 16, 18, 31 |  |
|  | 21 | 31 |  |
|  | 21 | 56 |  |
|  | 23 | 18 |  |
|  | 24 | 31 | 6 |
|  | 25 | 31, 56 |  |
|  | 26 | 16 |  |
|  | 27 | 31 |  |
|  | 28 | 59 |  |
|  | 29 | 16 |  |
|  | 30 | 56 |  |
|  | 31 | 56 |  |
|  | 32 | 51 |  |
|  | 33 | 39 |  |
|  | 34 | 31 |  |
|  | 35 | 58 |  |
|  | 36 | 56 |  |
|  | 37 | 31 |  |
|  | 38 | 35 | 6 |
|  | 39 | 39, 45 |  |
|  | 40 | 56 |  |
|  | 41 | 31, 39 |  |
|  | 42 | 31 |  |
|  | 43 | 56 |  |
|  | 44 | 16 |  |
|  | 45 | 59 |  |
|  | 46 | 45 |  |
|  | 47 | 16 |  |
|  | 48 | 16 |  |
|  | 49 | 16,18 |  |
|  | 50 | 31 |  |
|  | 51 | 52 |  |
|  | 52 | 39 |  |
|  | 53 | 56 |  |
|  | 54 | 52 |  |
|  | 55 | 39, 45 |  |
|  | 56 | 16 |  |
|  | 57 | 45 |  |
|  | 58 | 52 |  |
|  | 59 | 39 |  |
|  | 60 | 16 |  |
|  | 61 | 16/39/59 |  |
|  | 62 | 39/59 |  |
|  | 63 | 35 |  |
|  | 64 |  | 6 |
|  | 65 | 31, 45 |  |
|  | 66 | 51 |  |
|  | 67 | 51 |  |
|  | 68 | 31, 56 |  |
|  | 69 | 56 |  |
|  | 70 | 56 |  |
|  | 71 | 56 | 11 |
|  | 72 | 58 |  |
|  | 73 | 56 |  |
|  | 74 | 33 |  |
|  | 75 | 39 |  |
|  | 76 |  | 6 |
|  | 77 | 56 |  |
|  | 78 | 51 |  |
|  | 79 | 16, 39, 52 | 6 |
|  | 80 | 39 |  |
|  | 81 | 45 |  |
|  | 82 | 52 |  |
|  | 83 | 45 |  |
|  | 84 | 39 |  |
|  | 85 | 56, 58 |  |
|  | 86 | 45 |  |
|  | 87 | 18, 56 |  |
|  | 88 | 39 |  |
|  | 89 | 39, 58 |  |
|  | 90 | 51 |  |
|  | 91 | 16, 52 |  |
|  | 92 | 56 |  |
|  | 93 | 56 |  |
|  | 94 | 39, 52 |  |
|  | 95 | 45 |  |
|  | 96 | 59 |  |
|  | 97 | 31 |  |
|  | 98 | 56 |  |
|  | 99 | 31 |  |
|  | 100 | 18, 56 |  |
|  | 701 | 56 |  |
|  | 102 | 56 |  |
|  | 103 | 18 | 6 |
